# Extra-Nuclear Functions of the Transcription Factor Grainyhead-Like 3 in the Endothelium—Interaction with Endothelial Nitric Oxide Synthase

**DOI:** 10.3390/antiox10030428

**Published:** 2021-03-11

**Authors:** Kirsten Jander, Jan Greulich, Stefanie Gonnissen, Niloofar Ale-Agha, Christine Goy, Philipp Jakobs, Sabrina Farrokh, Corina Marziano, Swapnil K. Sonkusare, Judith Haendeler, Joachim Altschmied

**Affiliations:** 1 IUF-Leibniz Research Institute for Environmental Medicine, 40225 Düsseldorf, Germany; kirsten.jander89@gmx.de (K.J.); jan.greulich@hhu.de (J.G.); stefanie@gonnissen.de (S.G.); christinegoy100@posteo.eu (C.G.); sabrina.farrokh@googlemail.com (S.F.); 2 Environmentally-Induced Cardiovascular Degeneration, Clinical Chemistry and Laboratory Diagnostics, Medical Faculty, University Clinics, Heinrich-Heine-University, 40225 Düsseldorf, Germany; aleagha@hhu.de (N.A.-A.); philipp.jakobs@hhu.de (P.J.); 3 Robert M. Berne Cardiovascular Research Center, Department of Pharmacology, University of Virginia-School of Medicine, Charlottesville, VA 22908, USA; cm3xe@virginia.edu (C.M.); sks2n@virginia.edu (S.K.S.)

**Keywords:** grainyhead-like 3, endothelium, migration, apoptosis, endothelial NO synthase

## Abstract

We previously demonstrated that the transcription factor Grainyhead-like 3 (GRHL3) has essential functions in endothelial cells by inhibiting apoptosis and promoting migration as well as activation of endothelial nitric oxide synthase (eNOS). We now show that a large portion of the protein is localized to myo-endothelial projections of murine arteries suggesting extra-nuclear functions. Therefore, we generated various deletion mutants to identify the nuclear localization signal (NLS) of GRHL3 and assessed potential extra-nuclear functions. Several large-scale deletion mutants were incapable of activating a GRHL3-dependent reporter construct, which could either be due to deficiencies in transcriptional activation or to impaired nuclear import. One of these mutants encompassed a predicted bipartite NLS whose deletion led to the retention of GRHL3 outside the nucleus. Interestingly, this mutant retained functions of the full-length protein as it could still inhibit pathways inducing endothelial cell apoptosis. As apoptosis protection by GRHL3 depends on NO-production, we examined whether GRHL3 could interact with eNOS and showed a direct interaction, which was enhanced with the extra-nuclear GRHL3 variant. The observation that endogenous GRHL3 also interacts with eNOS in intact murine arteries corroborated these findings and substantiated the notion that GRHL3 has important extra-nuclear functions in the endothelium.

## 1. Introduction

The endothelium—the innermost layer of the vessel wall—is a single layer of cells that line the inside of blood vessels. They form a selective barrier between vessels and tissues and control the flow of substances into and out of a tissue. Nitric oxide (NO) produced by endothelial cells is essential for their functionality as well as for the maintenance of vascular homeostasis. In endothelial cells, NO is produced constitutively by the endothelial NO Synthase (eNOS). Impaired endothelial cell functionality is observed in nearly all cardiovascular diseases and is typically referred to as endothelial dysfunction. It is characterized by reduced NO bioavailability and migratory capacity as well as increased sensitivity towards apoptotic stimuli [1].

The transcription factor Grainyhead-like 3 (GRHL3) has been shown to be critical in wound healing and epidermal barrier function. It is also required for proper neural tube closure during embryonic development; therefore, GRHL3-deficient mice die shortly after birth due to an open back (spina bifida) [2,3]. Knockdown experiments in keratinocytes isolated from GRHL3-deficient mice revealed the involvement of GRHL3 in the migration of epidermal cells [4]. While mice express only a single GRHL3 protein, three different isoforms are found in humans, with isoform 2 being the direct homolog of the mouse protein. The two other isoforms are derived from a transcript with a different first exon that is not present in the mouse genome and an additional alternative splicing event, including or skipping exon 2; all other exons are identical in the three isoforms [5]. Previously, we found all GRHL3 isoforms to be expressed in primary human endothelial cells (EC) ex vivo [6,7]. There, isoforms 1 (GRHL3-1) and 2 (GRHL3-2) increased the migratory capacity and inhibited induction of apoptosis, whereas the N-terminally truncated isoform 3 (GRHL3-3) had opposite effects [6,7,8]. We have demonstrated that apoptosis protection by isoforms 1 and 2 and the pro-migratory effect of isoform 1 are NO-dependent. Although isoform 2 enhances endothelial migration and is critical for this process [6,8], the evidence for NO-dependent migration induction by this protein is only circumstantial. While it does not affect VEGF levels, it increases NO bioavailability by increasing phosphorylation of eNOS on serine 1177 [6] and endogenously produced NO has long been known to promote endothelial cell migration.

The underlying mechanisms leading to increased NO bioavailability and improved endothelial cell functionality are not completely understood. Moreover, the expression of GRHL3 in the vascular wall in vivo has not been studied so far. Therefore, the aim of this study was to analyze GRHL3 localization in murine arteries and to determine the mechanisms leading to improved endothelial cell functionality.

To our surprise, we found a relatively large portion of the transcription factor GRHL3 outside the nucleus in endothelial cells in vivo, mostly at myoendothelial projections, suggesting extra-nuclear functions. After delineation of the nuclear localization signal (NLS), we demonstrated that the extra-nuclear protein improves endothelial cell functionality. Moreover, GRHL3 interacted with eNOS outside the nucleus and thereby increased NO bioavailability, which probably explains the increased endothelial cell functionality.

In conclusion, GRHL3 is a new interaction partner of eNOS ex vivo and in vivo, and their interplay seems to be critical for vascular homeostasis and, thus, functions.

## 2. Materials and Methods

### 2.1. Cell Culture

Primary human endothelial cells (EC) were supplied from LONZA (Cologne, Germany), and human embryonic kidney cells (HEK293) were supplied from Invitrogen (Darmstadt, Germany). EC and HEK293 were cultured as previously described [9,10]. In detail, EC were cultured in endothelial basal medium supplemented with 1 μg/mL hydrocortisone, 12 μg/mL bovine brain extract, 50 μg/mL gentamicin, 50 ng/mL amphotericin B, 10 ng/mL epidermal growth factor (LONZA, Cologne, Germany), and 10% fetal bovine serum until the third passage. After detachment with trypsin, cells were grown for at least 20 h. HEK293 were cultured in DMEM GlutaMAX™ supplemented with 10% heat-inactivated fetal bovine serum and 1% penicillin/streptomycin.

### 2.2. Plasmids

To generate an expression vector for human full-length GRHL3 with a C-terminal myc-tag, the GRHL3 coding sequence from the original vector carrying a V5-tag [8] was amplified by PCR and inserted into pcDNA3.1/Myc-His(-) A (Invitrogen/Life Technologies, Darmstadt, Germany) opened with Xho I and Hind III using the Gibson Assembly^®^ Cloning Kit according to the manufacturer’s protocol (New England Biolabs, Frankfurt, Germany). Starting from this plasmid, the expression vectors for the GRHL3 deletion mutants were generated by Gibson Assembly. All constructs were verified by DNA sequencing. Cloning details and complete plasmid sequences are available upon request. The GRHL3-specific luciferase reporter plasmid has been described previously [6].

### 2.3. Transfection

Transient transfections with plasmid DNA in EC were performed using SuperFect (Qiagen, Hilden, Germany) as previously described [9,10]. In detail, EC were transfected on 6 cm culture dishes with 3 µg plasmid DNA and 22.5 µL SuperFect, or in 6-well plates with 1.2 µg plasmid DNA and 12 µL SuperFect per well. Transient transfections of HEK293 were performed using Lipofectamine^®^ 3000 transfection reagent (Thermo Fisher Scientific, Schwerte, Germany) according to the manufacturer’s instructions.

### 2.4. Migration Assays

Migration assays were performed with a scratch wound assay as previously described [11] or with a Boyden chamber Transwell migration assay. For detection of cell migration via scratch wound assay, wounds were set by scraping confluent cell monolayers with a sterile disposable rubber policeman. Therefore, endothelial cells were cultivated on 6 cm dishes, which were labeled with a trace line prior to setting the wound. After the injury, non-attached cells were removed by gentle washing with culture medium. The wound was created 5 h after transfection. Endothelial cell migration from the edge of the injured monolayer was quantified by staining the cells with 500 ng/mL 4′,6-diamidino-2-phenylindole (DAPI, Carl Roth, Karlsruhe, Germany) in PBS after the cells were fixed with 4% paraformaldehyde for 15 min at room temperature and microscopic pictures were taken using a Zeiss Axiovert 100. The cells, which had invaded the wound from the trace line, were automatically counted using the particle analysis feature of Image J 1.52a after watershed separation of overlapping nuclei.

For detection of cell migration via a Boyden chamber Transwell migration assay, cells were seeded and transfected on 6 cm dishes. 5 h after transfection, cells were detached with trypsin from the surface of the dishes and 2.6 × 10^5^ cells were transferred on Transwell inserts placed in 6-well plates (Corning Inc., Corning, NY, USA) containing culture medium with 20% fetal bovine serum, with etched coverslips on the bottom of the wells. Endothelial cell migration from the Transwell insert onto the edged coverslips was quantified by staining the migrated cells on the coverslips with 500 ng/mL DAPI (Carl Roth, Karlsruhe, Germany) in PBS after the cells were fixed with 4% paraformaldehyde for 15 min at room temperature and microscopic pictures were taken using a Zeiss Axiovert 100. The cells, which migrated through the Transwell insert, were automatically counted as described above.

### 2.5. Immunoblotting

After transfer of the proteins from polyacrylamide gels onto polyvinylidene difluoride membranes and blocking, membranes were incubated with antibodies directed against myc-tag (1:500), phospho-eNOS (S1177, 1:500) and Caspase-3 (1:3000 for full-length protein; 1:250 for the cleaved protein), all from Cell Signaling Technology, Frankfurt, Germany), GAPDH (1:70,000), eNOS (1:500) both from Abcam, Cambridge, UK, Topoisomerase I (1:200, Santa Cruz Biotechnology, Heidelberg, Germany), Tubulin (1:10,000; Sigma-Aldrich, Deisenhofen, Germany). Antibodies were incubated overnight at 4 °C. On the following day, membranes were incubated with secondary antibodies coupled to horseradish peroxidase, and detection was performed using ECL substrate (GE Healthcare, Solingen, Germany) and X-ray films.

### 2.6. Luciferase Reporter Gene Assay

Luciferase activity was measured as described [7]. In detail, after transfection, cells were lysed with Reporter Lysis Buffer (Promega, Mannheim, Germany) according to the manufacturer’s instructions. For HEK293, protein content was determined prior to the experiment, then a total of 10 µg protein was used. For EC, 10 µL of cell lysate was used, and protein content was determined and used for normalization.

### 2.7. S-NO Content

S-NO content in EC was measured as described previously [12,13] with one alteration: instead of 3.75 mM, p-chloromercuribenzosulfonic acid, 10 mM CuSO_4_ was used. In detail, cells were lysed in Griess lysis buffer (50 mM Tris-HCl, pH 8.0, 150 mM NaCl, 5 mM KCl, 1% Igepal CA630, 1 mM phenylmethylsulfonyl fluoride, 1 mM bathocuproinedisulfonic acid, 1 mM diethylenetriaminepenta-acetic acid, 10 mM N-ethylmaleimide), and 80 µg of cell lysate were incubated with 1% sulfanilamide and 0.1% N-(1-naphthyl)ethylenediamine in the presence or absence of 10 mM CuSO_4_ for 20 min. S-NO content was measured photometrically at 540 nm. The amount was calculated using defined GSNO concentrations as a standard.

### 2.8. Biochemical Fractionation

EC were lysed and fractionated using the NE-PER^TM^ Nuclear and Cytoplasmic Extraction Reagent according to the manufacturer’s protocol (Life Technologies, Darmstadt, Germany). The purity of the nuclear and cytosolic fractions was confirmed by immunoblotting with antibodies against Topoisomerase I (nuclear protein fraction) and GAPDH (cytosolic protein fraction).

### 2.9. Cyclic GMP Detection Assay

Cyclic GMP (cGMP) content in EC was determined using the cGMP Complete ELISA Kit according to the manufacturer’s instructions (Enzo Life Sciences, Lörrach, Germany). In brief, cells were lysed in 0.1 M HCl, and cell lysates, as well as cGMP conjugated to alkaline phosphatase, were added to the 96-well plate containing antibody against cGMP, binding the cGMP in the lysates and the conjugate competitively. After the addition of a substrate for the alkaline phosphatase, the absorbance was measured photometrically at 405 nm. The cGMP concentrations in the lysates were determined using the provided cGMP standards. All samples and standards were acetylated for increased sensitivity.

### 2.10. Immunostaining of EC

EC were fixed and permeabilized as described previously [11]. Cells were incubated with a primary antibody against the C-terminal myc-tag (1:50; Biomol, Hamburg, Germany) overnight at 4 °C and with an Alexa Fluor^®^ 488 coupled anti-mouse secondary antibody (1:500; Santa Cruz Biotechnology, Heidelberg, Germany) for 1 h at room temperature. Nuclei were stained with DAPI (500 ng/mL, Roche, Mannheim, Germany) for 5 min, and the actin cytoskeleton was stained with Phalloidin CF^®^ 488 (1:70; Biotium, Fremont, CA, USA) for 20 min. Images were taken using Zeiss microscopes (Axio Observer D1 or Axio Imager M2, magnification 40× oil).

### 2.11. Proximity Ligation Assay in EC

EC were seeded on acidified glass slides and transfected. 18 h after transfection, cells were fixed and permeabilized as described under immunostaining of EC. After overnight incubation at 4 °C with primary antibodies against the myc-tag or Akt1 (both: 1:100; Cell Signaling Technology, Frankfurt, Germany) and eNOS (1:100; BD Biosciences, Heidelberg, Germany), cells were treated with the components of the Duolink^®^ In Situ detection kit (Sigma-Aldrich, Deisenhofen, Germany) according to the manufacturer’s instructions. The actin cytoskeleton was stained with Phalloidin CF^®^ 488 1:70 for 20 min; nuclei were stained with DAPI 500 ng/mL for 5 min.

### 2.12. Immunostaining of Murine Mesenteric Arteries and Aorta

Immunostaining was performed on en face third-order mesenteric arteries or aorta as described previously [14,15]. In short, mesenteric arteries or thoracic aortas were isolated from 10–12 week old C57BL6 mice (Jackson Laboratories, Bar Harbor, ME, USA) and pinned down on a SYLGARD block. The arteries were fixed with 4% paraformaldehyde for 15 min at room temperature. Fixed arteries were washed three times for 5 min with phosphate-buffered saline (PBS). The arteries were then treated with 0.2% Triton-X/PBS for 30 min at room temperature on a rocker. Following this permeabilization step, arteries were treated with 5% normal goat serum (Abcam PLC, Cambridge, MA, USA) for 1 h at room temperature and subsequently incubated overnight with antibodies against GRHL3 (1:1,000; Merck Millipore, Darmstadt, Germany). Arteries were then washed three times with PBS and incubated with Alexa Fluor^®^ 568 goat anti-rabbit secondary antibody (1:500; Invitrogen/Life Technologies, Darmstadt, Germany) at room temperature for 1 h in the dark. Thereafter, arteries were washed three times with PBS and incubated with 0.3 μM DAPI (Invitrogen, Carlsbad, CA, USA) for 10 min at room temperature in the dark to stain nuclei. Images were obtained using the Andor Revolution WD (with Borealis) spinning-disk confocal imaging system (Andor Technology, Belfast, UK) comprising an upright microscope (Nikon, Tokyo, Japan) with a 60× water-dipping objective (numerical aperture, 1.0) and an electron-multiplying charge-coupled device camera, as described previously [16]. Consecutive images were taken along the z-axis at a slice thickness of 0.1 μm from the top surface of ECs to the bottom surface of SMCs. GRHL3 immunostaining was imaged by exciting at 561 nm and collecting the emitted fluorescence with a 607/36 nm band-pass filter. DAPI immunostaining was imaged by excitation with 409 nm and collecting the emitted fluorescence with a 447/60 nm band-pass filter. Autofluorescence of the internal elastic lamina was imaged using an excitation wavelength of 488 nm and a 525/36 nm band-pass emission filter.

### 2.13. Proximity Ligation Assay in Murine Mesenteric Arteries

Proximity ligation assay was performed on en face mesenteric arteries pinned on SYLGARD blocks as described previously [14]. Interaction between GRHL3 and eNOS was determined using primary antibodies against GRHL3 (as described above) and eNOS (1:100; BD Biosciences, Franklin Lakes, NJ, USA) and subsequently applying the DuoLink^®^ In Situ detection kit (Sigma-Aldrich, Deisenhofen, Germany) according to the manufacturer’s instructions.

### 2.14. Statistics

The number of experiments (n) given in figure legends represents independent biological replicates; the data shown are mean ± SEM. Normal distribution for all data sets was confirmed by Shapiro–Wilk test; homogeneity of variances (from means) between groups was verified by Levene’s test. Pairwise comparisons were performed with two-sided, unpaired Student’s *t*-tests on raw data. Multiple comparisons were performed using one-way ANOVA with post hoc Tukey’s LSD test.

## 3. Results

### 3.1. Localization of GRHL3 In Vivo

**Figure 1:**
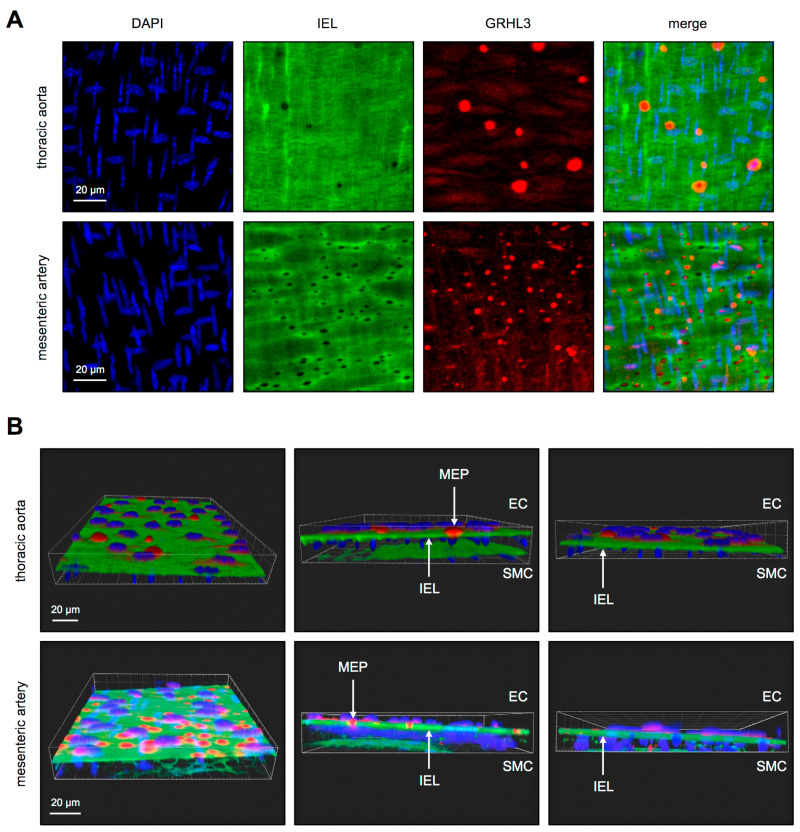
Transcription factor Grainyhead-like 3 (GRHL3) localization in the murine aorta and mesenteric arteries. (**A**) En face staining of the aorta (thoracic) and mesenteric arteries with an antibody against GRHL3 (red). Nuclei were counterstained with 4′,6-diamidino-2-phenylindole (DAPI) (blue). Horizontally-oriented nuclei are from endothelial cells and vertically oriented nuclei from smooth muscle cells. The internal elastic lamina (IEL) appears green due to autofluorescence. Black wholes in the IEL represent myoendothelial projections (MEP). Merge is the overlay of all channels. (**B**) Single frames from animated three-dimensional reconstructions of the aorta and mesenteric artery staining (see also Supplementary Videos). The endothelial cell layer (EC, top) is separated from the smooth muscle cell layer (SMC, bottom) by the internal elastic lamina (IEL). In the middle panels, MEPs are highlighted. Red staining represents GRHL3, which is only present in the endothelial cell layer, and blue shows the cell nuclei stained with DAPI.

We were the first to describe that the transcription factor GRHL3 is expressed in primary human endothelial cells ex vivo [6,7]. After generating an antibody against GRHL3 in cooperation with Merck Millipore, which is now commercially available, we performed en face staining of the murine aorta, which is a conduit artery, and resistance-sized mesenteric arteries to investigate whether GRHL3 is also expressed in endothelial cells in vivo. GRHL3 is detectable in the endothelium but not in the smooth muscle cell layer of those arteries (Figure 1). To our surprise, a large portion of the protein was localized outside the nucleus, which is unexpected for a transcription factor. Moreover, the majority of the extra-nuclear GRHL3 was found in myoendothelial projections (MEPs) (Figure 1 and Supplementary Videos). These MEPs are protrusions of endothelial cells reaching through the internal elastic lamina [17], thereby directly contacting the adjacent smooth muscle cells.

### 3.2. Identification of the Nuclear Localization Signal within Human GRHL3

On the basis of the unexpected finding that the transcription factor GRHL3 is also localized outside the nucleus and in MEPs, we hypothesized that GRHL3 has an up to now unknown non-nuclear function. To investigate this hypothesis, we first wanted to identify the NLS in GRHL3 to examine putative extra-nuclear functions of GRHL3 ex vivo, using the human isoform 2 that is the direct homolog of the mouse protein.

**Figure 2:**
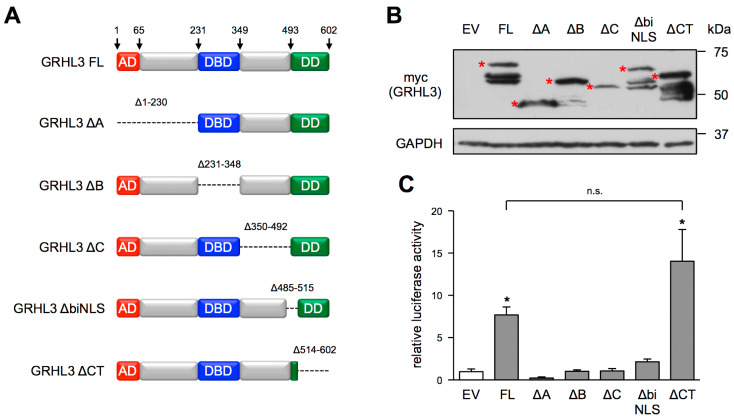
Transcriptional activity of full-length GRHL3 (GRHL3 FL) and of various deletion mutants in HEK293 cells. (**A**) Schematic representation of the GRHL3 variants ∆ denotes the deleted amino acids (AD = predicted activation domain; DBD = predicted DNA-binding domain; DD = predicted dimerization domain). (**B**,**C**) HEK293 cells were co-transfected with a GRHL3-specific luciferase reporter construct and expression vectors for the GRHL3 variants shown in (**A**). (**B**) Expression of the different GRHL3 variants was verified by immunoblot (upper panel, * marks proteins of the expected sizes), GAPDH served as a loading control (lower panel). (**C**) Luciferase activity was measured in cell lysates and is shown relative to cells co-transfected with an empty vector (EV) instead of GRHL3 variants (data are mean ± SEM, *n* = 4–10, * *p* < 0.05 vs. EV).

Therefore, we generated large-scale deletion mutants of GRHL3 (Figure 2A) based on the predicted functional domains [5] and our own previous experiments [7]. We then measured the transcriptional activation of a GRHL3-specific luciferase reporter [6] by these mutants in HEK 293 cells as this is strictly dependent on the nuclear localization of the transcription factor. In mutant ΔA (deletion of amino acids 1–230, Δ1–230), we deleted the predicted activation domain and the following sequences up to the DNA-binding domain, as we had previously shown that a splice variant that is translated into the N-terminally truncated isoform 3, which lacks the first 93 amino acids, but is still a fully active transcription factor [7]. Mutant ΔB (Δ231–348) lacks the DNA-binding domain (DBD) and thus, serves as a control, which is expected to be incapable of transcriptional activation. In ΔC (Δ350–492), we deleted the region between the DBD and a putative bipartite nuclear localization signal predicted by the software package cNLS Mapper [18], which is highly conserved between the human and the mouse protein and is missing in ΔbiNLS (Δ485–515). The last mutant, ΔCT (Δ514–602), does not contain the C-terminus of the protein with the majority of its dimerization domain (DD).

The full-length GRHL3 protein (FL) and ΔCT could activate the GRHL3-specific reporter to the same extent, suggesting that the dimerization domain is not essential for the transcription factor activity of GRHL3. In contrast, all other mutants, although expressed to similar levels (Figure 2B), did not show measurable transcription factor activity when compared to an empty vector, which was transfected as a negative control (Figure 2C).

This could be either due to a lack of an as yet unknown activation domain or to impaired nuclear import as a consequence of the deletion of the NLS, which could be the most likely explanation for the ΔbiNLS mutant.

**Figure 3:**
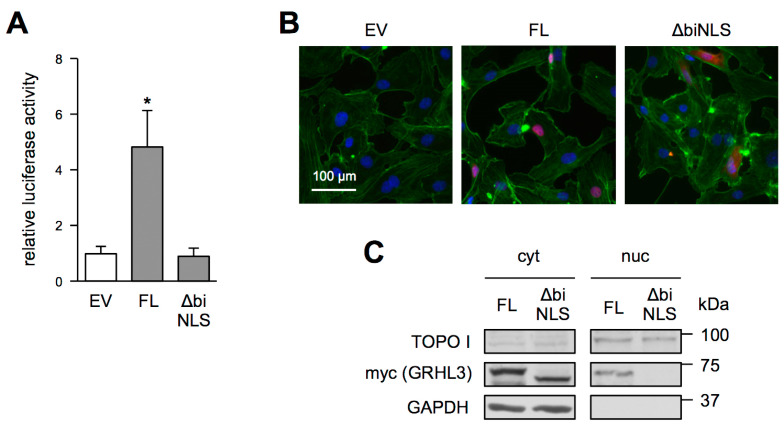
Transcriptional activity and intracellular localization of GRHL3 FL and GRHL3 ∆biNLS in EC. (**A**,**B**) EC were transfected with an empty vector (EV), GRHL3 FL (FL) or GRHL3 ∆biNLS (∆biNLS) plasmids. (**A**) Relative luciferase activity was measured in lysates of cells co-transfected with the GRHL3-specific luciferase reporter plasmid (data are mean ± SEM, *n* = 5, * *p* < 0.05 vs. EV). (**B**) Representative immunostainings. Nuclei were stained with DAPI (blue), GRHL3 FL and GRHL3 ∆biNLS were detected with an antibody against the myc-tag (red), the actin cytoskeleton was stained with Phalloidin (green). The merge of all channels is shown. (**C**) EC were transfected with GRHL3 FL (FL) or GRHL3 ∆biNLS (∆biNLS) plasmids. Representative immunoblots. Biochemical fractionation in cytosolic (cyt) and nuclear (nuc) fractions. Topoisomerase I (TOPO I, upper panels) and GAPDH (lower panels) served as purity controls of nuclear and cytosolic fractions, respectively. Myc represents the detection of the C-terminal myc-tag of GRHL3 FL and GRHL3 ∆biNLS (middle panels).

Assuming that GRHL3 ∆biNLS is retained in the cytosol, we next expressed this mutant and GRHL3 FL in EC and measured their transactivation potential with the same luciferase reporter as above. While the transcriptional activation by the full-length protein can easily be measured, EC expressing GRHL3 ∆biNLS showed no higher luciferase activity than cells transfected with an empty vector (Figure 3A). Therefore, we next determined the intracellular localization of GRHL3 FL and GRHL3 ∆biNLS. Immunostaining and biochemical fractionation revealed that GRHL3 ∆biNLS is only localized in the cytosol, clearly indicating that the deletion encompasses the NLS of GRHL3. In contrast, GRHL3 FL is detectable in the nucleus as well as in the cytosol (Figure 3B,C), confirming the results obtained in murine arteries (Figure 1).

### 3.3. Role of Extra-Nuclear GRHL3 in Endothelial Cell Functionality, NO Bioavailability, eNOS Phosphorylation and Interaction of Akt1 and eNOS

**Figure 4:**
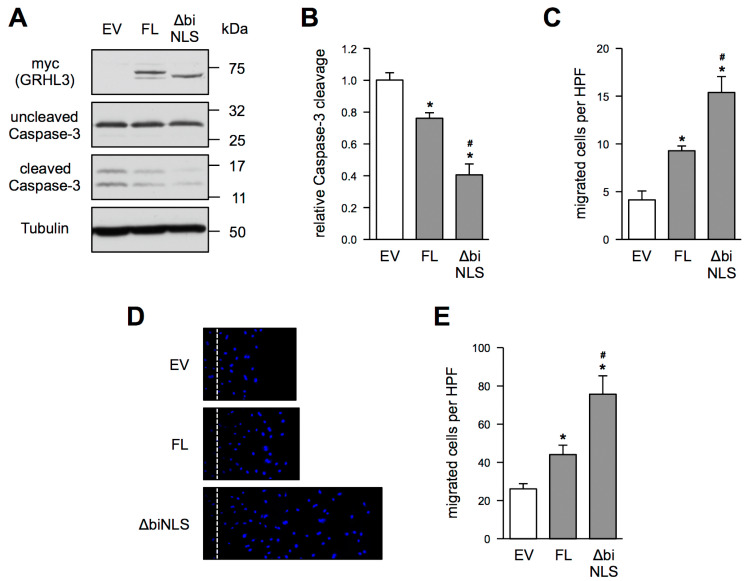
Effects of GRHL3 FL and GRHL3 ∆biNLS on apoptosis and migration in EC. (**A**–**E**) EC were transfected with an empty vector (EV), GRHL3 FL or GRHL3 ∆biNLS plasmids. (**A**) Representative immunoblot. Myc represents the expression of GRHL3 FL, and GRHL3 ∆biNLS (upper panel), uncleaved Caspase-3 (upper, middle panel), cleaved Caspase-3 (lower, middle panel), Tubulin served as a loading control (lower panel). (**B**) Semiquantitative analyses of the relative amount of cleaved Caspase-3 (data are mean ± SEM, *n* = 6, * *p* < 0.05 vs. EV, # *p* < 0.05 vs. FL). (**C**) Migratory capacity was measured in a Boyden chamber assay. Image J analyses of migrated cells per high-power field (HPF) (data are mean ± SEM, *n* = 5, * *p* < 0.05 vs. EV, # *p* < 0.05 vs. FL). (**D**,**E**) Migratory capacity was measured in a scratch-wound assay. (**D**) Representative DAPI staining. Wounds were set at the scattered lines; left of the lines are the unwounded areas. The areas right to the lines show the wounds with the cells that had migrated into them. Nuclei were stained with DAPI. (**E**) Image J analyses of migrated cells per high-power field (HPF) (data are mean ± SEM, *n* = 5, * *p* < 0.05 vs. EV, # *p* < 0.05 vs. FL).

To understand the role of extra-nuclear localized GRHL3 in EC, we compared GRHL3 ∆biNLS and GRHL3 FL in their ability to inhibit apoptosis and to enhance migratory capacity. We had previously demonstrated that GRHL3 inhibits apoptosis and enhances migration [6,7], both in a NO-dependent manner. We had also shown that GRHL3 enhances NO bioavailability and the phosphorylation of eNOS on serine 1177 [6,7]. Therefore, EC were transfected with an empty vector, GRHL3 FL or GRHL3 ∆biNLS plasmids. First, comparable overexpression of GRHL3 FL and GRHL3 ∆biNLS in EC was controlled by immunoblotting (Figure 4A). Then, apoptosis was measured using the amount of cleaved Caspase-3 as a standard marker. GRHL3 FL inhibited apoptosis as published previously by us [6,7]. Interestingly, the extra-nuclear localized GRHL3 ∆biNLS blocked apoptosis more pronounced than GRHL3 FL (Figure 4A,B). Next, we investigated the migratory capacity of EC using a Boyden chamber and a scratch wound assay. As shown in Figure 4C–E, GRHL3 FL enhanced migration of EC when compared to empty vector-transfected cells; however, migration was enhanced even stronger when GRHL3 ∆biNLS was expressed. Thus, the functionality of endothelial cells is even more improved when GRHL3 is outside of the nucleus. Since apoptosis protection and migratory capacity of endothelial cells depend on the bioavailability of NO, we next measured the S-NO content, which is used as a surrogate marker for NO bioavailability [10].

**Figure 5:**
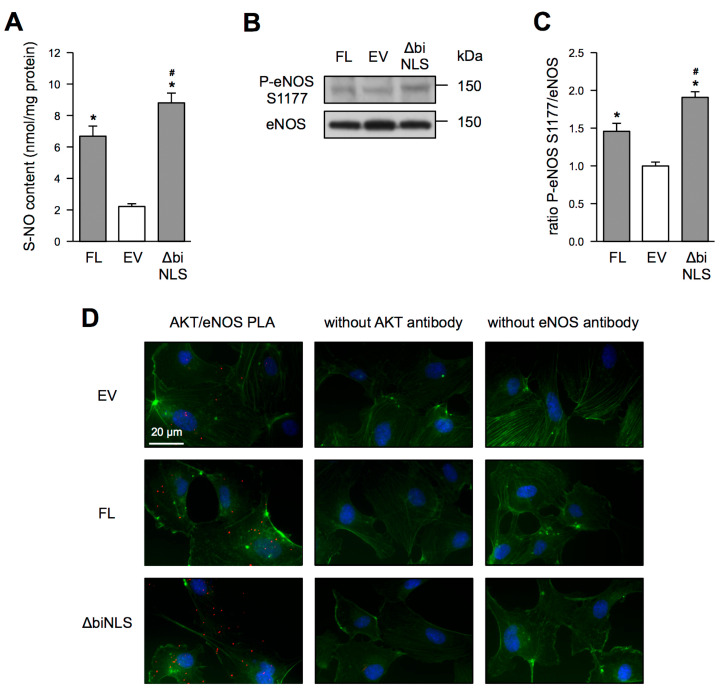
Effects of GRHL3 FL and GRHL3 ∆biNLS on NO bioavailability, eNOS phosphorylation and interaction of Akt1 and eNOS. (**A**–**D**) EC were transfected with an empty vector (EV), GRHL3 FL or GRHL3 ∆biNLS plasmids. (**A**) S-NO content was measured at 405 nm and calculated as nmol/mg protein (data are mean ± SEM, *n* = 5, * *p* < 0.05 vs. EV, # *p* < 0.05 vs. FL). (**B**,**C**) Phosphorylation of eNOS on serine 1177 was measured by immunoblot. (**B**) Representative immunoblot. The upper panel shows phosphorylation of eNOS on serine 1177. The lower panel shows comparable levels of total eNOS. (**C**) Semiquantitative analyses of the ratio of phosphorylated eNOS on serine 1177 to total eNOS (data are mean ± SEM, *n* = 5, * *p* < 0.05 vs. EV, # *p* < 0.05 vs. FL). (**D**) Proximity ligation assay (PLA) was performed with antibodies against AKT and eNOS (AKT/eNOS PLA, left panels). As negative controls, PLAs were performed, omitting either the AKT antibody (middle panels) or the eNOS antibody (right panels). Red dots represent AKT and eNOS interactions. The actin cytoskeleton was stained with Phalloidin (green) and nuclei with DAPI (blue).

In agreement with the improved endothelial cell functionality in EC overexpressing GRHL3 ∆biNLS, this extra-nuclear GRHL3 variant increased the S-NO content significantly when compared with GRHL3 FL (Figure 5A). Therefore, we also determined whether GRHL3 ∆biNLS improves phosphorylation of eNOS on serine 1177, which activates the enzyme. As expected and previously published by us, GRHL3 FL enhanced phosphorylation of eNOS on serine 1177 (Figure 5B) [6,7]. Corroborating the S-NO content data, GRHL3 ∆biNLS overexpressing EC showed significantly more phosphorylation on serine 1177 when compared to GRHL3 FL (Figure 5B,C). This increase in intracellular, bioavailable NO could result in increased cyclic GMP (cGMP) levels. However, cGMP measurements revealed no differences in EC transfected with empty vector, GRHL3 FL and GRHL3 ∆biNLS plasmids (EV: 0.183 +/− 0.04 pmol/mL, GRHL3 FL: 0.155 +/− 0.04 pmol/mL, GRHL3 ∆biNLS: 0.154 +/− 0.02, data are mean ± SEM, *n* = 3–4, n.s.). An increase in eNOS phosphorylation on serine 1177 could also be explained by an increase in the interaction between Akt1 and eNOS. Therefore, we performed proximity ligation assays for Akt1 and eNOS in EC transfected with an empty vector or the GRHL3 expression plasmids. As demonstrated in Figure 5D, the interaction between Akt1 and eNOS is more pronounced in cells overexpressing GRHL3 ∆biNLS than in GRHL3 FL overexpressing EC.

### 3.4. GRHL3 eNOS Interaction Ex Vivo and In Vivo

**Figure 6:**
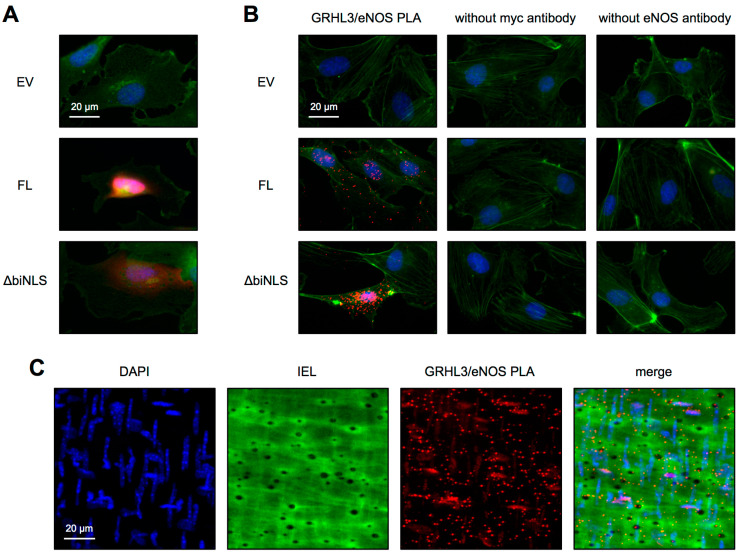
GRHL3 and eNOS interaction ex vivo and in vivo. (**A**,**B**) EC were transfected with an empty vector, GRHL3 FL or GRHL3 ∆biNLS plasmids. (**A**) Representative immunostainings. Co-immunostainings with an anti-myc-tag antibody and an eNOS antibody were performed. Myc-tag represents GRHL3 variants (red), eNOS is shown in green, nuclei were stained with DAPI (blue). (**B**) Proximity ligation assay (PLA) was performed with an antibody against the myc-tag and an eNOS antibody (GRHL3/eNOS PLA, left panels). As negative controls, PLAs were performed, omitting either the myc antibody (middle panels) or the eNOS antibody (right panels). Red dots represent eNOS and GRHL3 interactions. The actin cytoskeleton was stained with Phalloidin (green) and nuclei with DAPI (blue). (**C**) Proximity ligation assay (PLA) in the mesenteric artery using GRHL3 and eNOS antibodies. Nuclei were counterstained with DAPI. Horizontally-oriented nuclei are from endothelial cells, and vertically oriented nuclei are from smooth muscle cells. Shown are nuclear stains with DAPI (blue). The autofluorescence of the internal elastic lamina (IEL) is shown in green. Black holes in the IEL represent myoendothelial projections (MEP). Red dots represent eNOS and GRHL3 interactions. Merge is the overlay of all channels.

The S-NO content in EC depends on the production of NO by eNOS, and eNOS is mainly localized outside the nucleus and notably also in MEPs [16]. Therefore, we hypothesized that GRHL3 and eNOS could be in close proximity to each other and that one extra-nuclear function of GRHL3 is the interaction with eNOS resulting in activation of the enzyme and, thus, increased NO bioavailability. To test this hypothesis, we first performed immunostainings in EC overexpressing GRHL3 FL or GRHL3 ∆biNLS using antibodies against eNOS and the myc-tag to specifically detect GRHL3 FL as well as GRHL3 ∆biNLS. As shown in Figure 6A, GRHL3 and eNOS colocalize. To further investigate whether GRHL3 and eNOS are in so close proximity that they interact with each other, proximity ligation assays were performed. Indeed, GRHL3 and eNOS interact with each other (Figure 6B). Interestingly, the interaction between eNOS and GRHL3 ∆biNLS is more pronounced than the interaction between eNOS and GRHL3 FL. Thus, one could assume that the increase in S-NO content shown in Figure 5A is due to enhanced interaction between extra-nuclear GRHL3 and eNOS.

Finally, we also performed in situ proximity ligation assays in the murine mesenteric arteries using antibodies against eNOS and GRHL3 to determine whether our results in cells also hold true in vivo. As shown in Figure 6C, GRHL3 also showed nanometer proximity with eNOS in the endothelium of murine mesenteric arteries in situ.

Thus, GRHL3 is a new interaction partner for eNOS ex vivo and in vivo, and its extra-nuclear localization leads to enhanced endothelial cell functionality, probably by improving NO production.

## 4. Discussion

The major finding of the present study is that the transcription factor GRHL3 has an extra-nuclear function in primary human endothelial cells. Extra-nuclear GRHL3 interacts with eNOS in EC and murine arteries. It improves NO bioavailability and migratory capacity and inhibits apoptosis.

Transcription factors normally exert their function by binding to DNA and activating their target genes within the nucleus. However, several studies have demonstrated that transcription factors can have functions outside the nucleus independent of target gene activation. In contrast to GRHL3, which inhibits apoptosis in endothelial cells, the transcription factor p53 has been shown to induce apoptosis upon DNA damage. Within the nucleus, p53 induces the expression of target genes leading to apoptosis induction. However, p53 also has extra-nuclear functions. In the cytoplasm, it induces the release of Cytochrome C from the mitochondria into the cytosol, which increases the apoptotic process [19]. Thus, the activation of target genes by p53 and its extra-nuclear function are both intimately involved in apoptosis induction. Along the same lines, but with opposite outcome in endothelial cells, nuclear GRHL3-1 induces expression of Bcl-X_L_ [7], an anti-apoptotic protein, which protects the outer mitochondrial membrane from damage, among other organs also in the cardiovascular system [20]. Here, we demonstrate that extra-nuclear GRHL3-2 enhances the bioavailability of NO and, thus, inhibits apoptosis. Due to the very short coding part of the two different first exons in the transcripts coding for GRHL3-1 and GRHL3-2, these proteins differ only in very few amino acids at their extreme N-terminus, with the first 11 amino acids in isoform 1 being different from the first six amino acids in isoform 2, while the remainder of the two proteins is identical. Thus, it seems reasonable to assume that both protective isoforms of GRHL3 function in the same manner. Therefore, one could hypothesize that activation of gene transcription by GRHL3-1 and GRHL3-2 as well as their extra-nuclear function are both required for apoptosis protection. It is also worth speculating that, dependent on the external stimuli hitting the endothelium, fast protection could be required, and under such circumstances, GRHL3-2, and most likely also GRHL3-1, is exported from the nucleus to increase the GRHL3/eNOS interaction and thus, NO bioavailability. Increased intracellular NO would lead to inhibition of, e.g., Caspase-3 activation, since increased NO leads to enhanced S-nitrosation of cysteine 163 in Caspase-3, which prevents the generation of the fully active enzyme [21]. This is in line with findings in our study here, demonstrating that increased extra-nuclear GRHL3 leads to higher S-NO content and to reduced cleaved Caspase-3. Besides an increase in the S-NO content, we also found enhanced active phosphorylation of eNOS and interaction of Akt1 and eNOS. Thus, one could assume that the resulting increase in intracellular NO would lead to S-nitrosation of several proteins besides Caspase-3, which are important in apoptosis protection, like, for instance, Thioredoxin-1 [9,10]. However, further studies are required to determine which S-nitrosated proteins may be involved in apoptosis protection conferred by GRHL3 in EC.

Another example of the interplay between nuclear and extra-nuclear GRHL3 is the induction of migration. We have previously published that GRHL3-1 activates transcription and protein expression of Akt2. Akt2, as a master regulator of other Akt isoforms, phosphorylates Akt1, which in turn activates eNOS [7]. Moreover, Akt2 is intimately connected to enhanced cell migration [22]. On the other hand, we demonstrate here that isoform 2 lacking the NLS has a more pronounced effect on migration than the full-length protein, which must be independent of transcription. Thus, one could hypothesize that, as eluded to above, the induction of endothelial cell migration by GRHL3-1 and GRHL3-2 requires concerted action inside and outside the nucleus.

It must be noted that the enhanced effects of GRHL3 ΔbiNLS on the different outputs in EC could simply be due to the fact that all of the overexpressed protein is excluded from the nucleus. Thereby, the cytosolic concentration of GRHL3 is higher than when the full-length protein is expressed such that the extra-nuclear functions are more pronounced.

We demonstrate here for the first time that GRHL3 interacts with eNOS. It has been demonstrated that eNOS forms a complex with the Heat-shock protein 90 (Hsp90). The binding of Hsp90 to eNOS enhances the activation of eNOS [23], most likely through phosphorylation on serine 1177 by Akt1. Since Hsp90 is a chaperone, it is tempting to speculate that Hsp90, eNOS, Akt1 and GRHL3 form a larger complex and that GRHL3 contributes to increased activation of eNOS. However, further studies are needed to delineate the nature of the interactions between those proteins.

Another new finding of our study is the presence of GRHL3 in MEPs. MEPs are protuberances of the plasma membrane of the endothelium to generate close contact to the subjacent smooth muscle cells. Recently, it has been demonstrated that eNOS is also localized in MEPs. Within those MEPs, eNOS cooperates with Transient Receptor Potential Vanilloid 4 (TRPV4) ion-channels to enhance vasodilation. The increase in calcium ions by the TRPV4 ion-channels leads to activation of eNOS and NO production [16]. Thus, one could speculate that the interaction of eNOS with GRHL3 also in these MEPs will further enhance NO production and, thus, contribute to vasodilation.

## 5. Conclusions

In conclusion, our study shows for the first time that the transcription factor GRHL3 has extra-nuclear functions. Moreover, GRHL3 is a new interaction partner of eNOS in EC and in murine arteries. An increase in extra-nuclear GRHL3 results in enhanced migratory capacity and NO bioavailability, and reduced apoptosis. Thus, not only the transcriptional activity of GRHL3 but also its extra-nuclear actions contribute to improved functionality of endothelial cells.

## Data Availability

Raw data will be made available by the corresponding authors upon reasonable request.

## Supplementary Materials

The following are available online at https://www.mdpi.com/2076-3921/10/3/428/s1, Video S1: 3D reconstruction aorta, Video S2: 3D reconstruction mesenteric artery.
